# Predicting the Need for Fluid Therapy—Does Fluid Responsiveness Work?

**DOI:** 10.1186/s40560-017-0210-7

**Published:** 2017-06-06

**Authors:** Hiroshi Ueyama, Sawami Kiyonaka

**Affiliations:** Department of Anesthesiology, Kansai Rosai Hospital, 3-1-69, Inabaso, Amagasaki, 660-8511 Hyogo Japan

**Keywords:** Fluid responsiveness, Fluid challenge, Starling’s law, Starling mechanism, Crystalloid, Colloid, Preload, Afterload, Systemic vascular resistance, Cardiac output, Stroke volume, Fluid therapy

## Abstract

Fluid overdose can be harmful in critically ill patients. Since central venous pressure (CVP) is currently considered to be an inappropriate indicator of preload, much attention is being given to predicting fluid responsiveness, i.e., the response of stroke volume (SV) or cardiac output (CO) to fluid challenge. However, when fluid responsiveness was evaluated in critically ill patients, including sepsis, only 40–50% of the patients responded. Moreover, most fluid responders do not show significant hemodynamic improvement after fluid administration. In this review, we discuss why fluid responsiveness based on the Starling mechanism did not work well in the clinical setting.

According to the Starling mechanism, a patient whose SV/CO significantly increases after a fluid challenge is considered to be a fluid responder and judged to need fluid therapy. However, the currently recommended fluid challenge dose of crystalloid 250–500 mL has little effect on increasing blood volume and is not sufficient to increase the preload of the Starling curve. Especially in septic patients, due to their vascular hyperpermeability, increase in blood volume is even smaller. Furthermore, Infusion induced hemodilution is known to reduce blood viscosity and hematocrit, as a result, decreasing afterload. This indicates that the increased SV/CO after fluid challenge is caused not only by increased preload but also by decreased afterload. For these reasons, fluid responsiveness with small crystalloid challenge is questionable as a clinical indicator of fluid therapy.

## Background

Fluid therapy has been used to prevent or to treat circulatory failure. However, excessive fluid in critically ill patients has been recognized to cause cardiac complications, including pulmonary edema and heart failure [1, 2]. It is especially important for septic patients and for those with adult respiratory distress syndrome (ARDS) to discriminate which patients are expected to have improved hemodynamics with fluid therapy.

The venous blood is theoretically separated into stressed and unstressed volume [3]. The unstressed volume is defined as the blood volume necessary to fill the venous system, and only the stressed volume, i.e., volume that surpasses the unstressed vein, refluxes to the heart and contributes to cardiac output (CO). In healthy patients, 70% of the venous blood is unstressed volume and 30% is stressed volume [3]. The rational for the necessity of fluid therapy for septic patients is that they are relatively hypovolemic due to blood retention in their unstressed volume from venodilation [3]. Hence, CO is improved by the correction of blood volume.

Hemodynamic monitoring has been used to guide fluid therapy. Central venous pressure (CVP) has been used as an indicator for fluid therapy for a long time. Since the compliance of the vein is 30 times higher than that of the artery [4], CVP varies little with changes in blood volume; hence, it is an inappropriate indicator of venous volume [5].

Instead of CVP, attention is being paid to the assessment of fluid responsiveness [4]. Fluid responsiveness is a relatively new concept evaluating the need for the fluid therapy, by checking the response of stroke volume (SV) or CO to fluid challenge, in accordance with the Starling mechanism [6]. However, when the fluid responsiveness was evaluated in critically ill patients, including sepsis, only 40–50% of the patients responded [7]. This result suggests that only half of the critically ill patients needed fluid therapy. More importantly, most fluid responders do not show significant hemodynamic improvement after fluid administration [8–10]. This review discusses the problems with fluid responsiveness based on the Starling mechanism.

## Review

### Starling Curve and Fluid Responsiveness

**Fig. 1:**
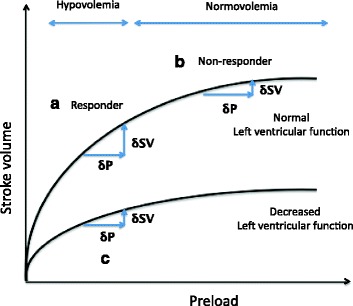
Starling curve and left ventricular function: the relationship between SV and preload. **a** If fluid challenge adequately improves SV, a patient is considered hypovolemic (responder). **b** If fluid was given at the plateau of Starling curve, SV will not increase, and a patient is considered normovolemic (non-responder). **c** For decreased left ventricular function cases, SV response after fluid loading cannot be observed even in hypovoelia, as Starling curve is flatter than the normal cardiac function cases. *SV* stroke volume

In the 1920s, E.H. Starling, an English physiologist, conducted a study on the heart and lungs of dogs and showed that the CO increased as the right atrial filling pressure increased by elevating the height of the venous blood reservoir [6]. He also showed that the CO conversely decreased after the right atrial filling pressure increased beyond a certain point (Fig. 1). The device used in this study used artificial aortic resistance. Since arterial resistance was constant in almost all cases, CO correlates with atrial filling pressure [6].

The fluid responsiveness can be explained using the Starling curve as follows [11].

1. If rapid fluid challenge improves SV/CO, the fluid responsiveness is considered positive. The patient is considered hypovolemic, and both CO and tissue perfusion are expected to increase by fluid therapy (Fig. 1a).
2. If SV is not improved by fluid challenge, the fluid responsiveness is considered negative. Aggressive fluid therapy in this situation increases the risks for both pulmonary edema and heart failure (Fig. 1b).
3. If left ventricular function is decreased, the SV/CO response is minimal even if the preload is increased, consequently, the above interpretation cannot be applied, (Fig. 1c).

**Fig. 2:**
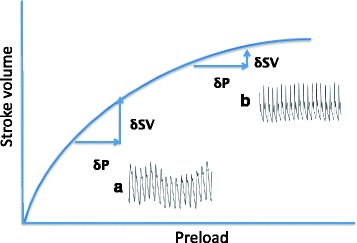
Starling curve and respiratory variation of SV. At the point where the ascending limb of Starling curve, respiratory variation in the stroke volume is significant (**a**) and judged as a large preload reserve. While at the point where the Starling curve is nearly flat, the respiratory variation is minimal (**b**) with a small preload reserve. *SV* stroke volume

The SV/CO changes due to fluid challenge have been evaluated using both pulmonary artery catheterization and Doppler transesophageal echocardiography. Recently, devices to measure stroke volume variation (SVV) and pulse pressure variation (PVV) have been developed to evaluate fluid responsiveness [11]. SVV and PVV are based on mechanical ventilation-induced changes in preload resulting in respiratory variations in SV or arterial pressure, respectively. The fluid responsiveness is evaluated with these parameters using the findings that SVV/PVV is greatly increased at the ascending limb of the Starling curve (Fig. 2a), while SVV/PVV is minimal at the point where the Starling curve reaches a plateau (Fig. 2b). Although these devices are expensive, they are easy to use and have been widely used not only for control of critically ill patients but also for surgical patients.

### Problems in Applying the Starling Curve to Monitoring Fluid Responsiveness

Fluid challenge with 6 mL/kg (250–500 mL) of crystalloid for 15 min is currently recommended, and patients with an SV increase of 10–15% are determined to be fluid responders [12]. However, the use of the Starling curve as an indicator of fluid responsiveness has not been fully validated due to the following problems.

1. Dose the right atrial filling pressure increase in parallel with the infused volume?

To explain the fluid responsiveness using the Starling curve, preload or right filling pressure is used as the horizontal axis and stroke volume as the longitudinal axis. However, except for patients after cardiac surgery, the right filling pressure cannot be measured. Fluid responsiveness was evaluated under the assumption that the preload was increased by the fluid challenge. But, it is not clear whether the administered fluid increases preload in a volume-dependent manner.

A clinical study showed that when 1.5 L of lactated Ringer’s solution was infused for 30 min prior to cesarean delivery, only 25% of the infused solution remained in the blood and the fluid increased the blood volume only by 7% [13]. Since blood volume in humans generally ranges from 4 to 6 L, the expansion effect of 500 mL of lactated Ringer’s solution is estimated as maximum 200 mL immediately after fluid challenge. This volume increase is considered minimal relative to the total blood volume. Therefore, the 250–500 mL of crystalloid fluid challenge, which is currently recommended for evaluation of fluid responsiveness, is not sufficient to increase preload and the right atrial pressure of the Starling curve.

**Fig. 3:**
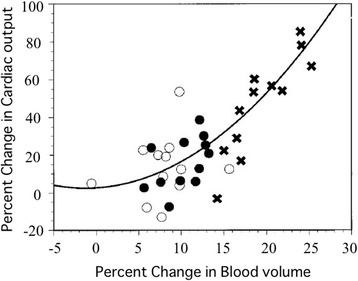
The relation between percent change in blood volume and cardiac output (CO) after volume preload with 1.5 L lactated Ringer’s solution (〇), 0.5 L hydroxyethylstarch solution, 6% (●), and 1.0 L hydroxyethylstarch solution, 6% (×) in parturients at term. [15]. Exponential increase in CO was observed after volume preloading

Furthermore, individual differences in the effect of infusion on blood volume have also been found. Svensén et al. administered 25 mL/kg of lactated Ringer’s solution to patients who underwent abdominal surgery for 45 min during surgery and found that 40% (4/10) of the patients were fluid responders in whom the CO increased, and the others were non-responders [14]. Ueyama et al. also confirmed the range of blood volume change using 1.5 L lactated Ringer’s solution, 0.5 or 1.0 L of hydroxyethyl starch (HES) to be 0–10, 5–13, and 15–25%, respectively, which reflects individual differences [13] (Fig. 3).

Sepsis is characterized by diffuse endothelial injury and shedding of the endothelial glycocalyx layer, which induces capillary hyperpermeability. Consequently, crystalloid and colloid solutions cannot be expected to remain in the intravascular space of septic patients. Studies have shown that only 5% or less of the crystalloid infusion remained in the intravascular volume after 1 h in septic patients [15, 16].

In summary, the blood volume expansion effect of 250–500 mL of crystalloid is not only insufficient but also variable among individuals, so this menu is inappropriate for fluid challenge. Furthermore, the mathematical effect of fluid challenge does not translate to septic patients because the blood volume expansion effect of fluid was less in septic patients. Therefore, problems remain with the use of the SV/CO response to small amounts of infusion.

1. Decreased Afterload by Fluid-Induced Hemodilution

CO is affected not only by preload but also by afterload. In Starling’s experiment, the blood was used as preload. However, unlike the blood, fluid reduces afterload, i.e., systemic vascular resistance (SVR) by hemodilution.

Infusion is considered to decrease SVR due to the following effects on blood flow characteristics.

- Hemodilution decreases blood viscosity and hematocrit.
- When the blood is diluted, the thickness of the plasma layer in the arteriole increases, leading to decreased vascular resistance with the arteriolar wall [17, 18].

When colloid was administered to patients who underwent peripheral vascular surgery, the CO actually increased but the SVR and mean arterial pressure (MAP) decreased [19].

In septic patients, hemodilution due to volume preload also induced a decrease in SVR. Monge-Garcia et al. administered 500 mL of either crystalloid or colloid for 30 min and evaluated the SV/CO using Doppler transesophageal echocardiography and reported a 10% decrease in the SVR by infusion in fluid responders (67%) [10]. This result suggests that not only increased preload but also decreased afterload is involved in the increase of SV/CO with small amounts of fluid challenge. Marik et al. stated “ Fluid boluses should be considered vasodilator therapy in patients with sepsis and that aggressive fluid resuscitation may potentiate the hyperdynamic state.” [20].

Approximately 50% of septic patients develop myocardial damage caused by inflammatory cytokines and by vascular hyperpermeability earlier at onset, and cardiac function decreases at the early stage of sepsis [21]. In the Starling curve, if left ventricular function is low, the SV/CO is considered not to increase despite the fluid administration in hypovolemic conditions (fig. 1c). However, if afterload is decreased by hemodilution due to fluid administration, the SV/CO may increase despite the low left ventricular function.

Hence, decreased afterload due to hemodilution has a large impact on the interpretation of the fluid responsiveness in fluid challenge.

1. Does infusion increase SV/CO similar to the Starling curve?

**Fig. 4:**
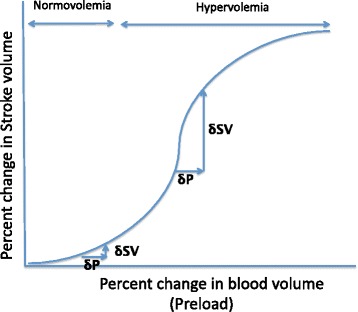
Schematic illustration of the hypothetical Starling curve after fluid administration. Infusion beyond normovolemia may exponential increase SV until the onset of heart failure. The maximum increase in SV against preload change (δSV/δP) may observe at hypervolemic state

If fluid administration behaved like the Starling curve, the increase in blood volume by infusion should linearly increase CO. However, this does not agree with the past findings that evaluated the relationship between percent change in blood volume and in CO after crystalloid or colloid administration in parturients [13]. This result showed that 8 to 10% increases in blood volume produced by 1.5 L of lactated Ringer’s solution, and 0.5 L of HES were associated with 11 to 14% increase in CO. However, doubling the increase in blood volume (20%) by 1.0 L of HES more than tripled (43%) CO (Fig. 3). As this marked increase in CO in the 1 L HES group was associated with 20% decrease in hemoglobin (Hb) value, the increase in CO is probably due to not only an increase in preload but also a decrease in afterload. This result suggested that SV/CO may increase exponentially, not linearly, as the dose of fluid is increased, even in the normovolemic or hypervolemic state if cardiac function is preserved (Fig. 4). Therefore, it is inappropriate to apply the SV/CO changes by fluid challenge to the conventional Starling curve.

### Effect of Fluid Therapy on Blood Pressure and Blood Volume

One of the objectives of fluid therapy for critically ill patients is to increase both blood pressure and tissue perfusion pressure by increasing the CO. MAP is defined as follows.

MAP (mmHg) = SVR (dyne*sec*cm^−5^) × CO (L/min) ÷ 80

This equation shows that blood pressure is the product of SVR and CO.

This equation also shows that for increase in blood pressure, changes in SVR and CO need to maintain a good balance. If fluid is given to a patient with low blood pressure due to bleeding, the reduced CO increases if the blood volume is sufficiently increased beyond the unstressed volume, which leads to increase in blood pressure. However, even if blood pressure is increased by fluid therapy, it is almost impossible to increase systolic blood pressure to 100 mmHg or higher with infusion alone. The reason is that increased CO by fluid administration is offset by the decreased SVR due to hemodilution. Conditions are consistent with blood pressure control for critically ill patients, who have sepsis or ARDS. SVR is also known to decrease by infusion in septic patients [10]. Furthermore, in septic patients, although the MAP is transiently increased by the fluid bolus administration, it returns to base line within 1 h [8, 9].

This result suggests that, in critically ill patients, the effect of the fluid therapy on blood pressure is minimal and the duration is short. Therefore, not only fluid therapy but also vasopressors, including noradrenaline, is necessary to increase vascular resistance and subsequently increase blood pressure.

If fluid responsiveness using the SV/CO changes does not work well, how should we evaluate the effect of fluid therapy on blood volume? A simple method to detect the change in blood volume after fluid administration is the measurement of Hb value. For example, in the situation without bleeding, the decrease in Hb value from 10 to 9 g/dl after fluid administration indicates approximately a 10% increase in blood volume. In a clinical study, the changes in Hb value after fluid administration inversely related with increase in blood volume [13]. If the decreased Hb value after fluid administration increases with time, it means that blood volume augmentation effect of fluid has disappeared.

Rehm et al. recorded the in-out balance and blood volume before and after gynecologic surgery in 18 patients. This study found that the mean blood volume decreased from approximately 5100 mL before surgery to approximately 4600 mL after surgery although transfusion and infusion with colloid were performed for intraoperative bleeding and the intraoperative in-out balance was +3800 mL [22]. Similar results were also found in patients who underwent cardiac and neurosurgical surgery. These results suggest that nearly all infused solutions do not remain in the blood vessels and probably move to interstitial tissues, regardless of whether crystalloid or colloid is administered.

### Fluid responsiveness and prognosis

Since the introduction of early goal-directed therapy (EGDT) in 2001 by Rivers et al. [23], the mortality of sepsis decreased. As this EGDT regimen included a fluid therapy, fluid administration is thought to be essential. However, recent clinical study in septic patients showed that positive fluid balance at initial 12 h and day 4 is associated with higher mortality [2]. Furthermore, recent multicenter clinical studies (ProCESS, ARISE, and PROMISE) showed that less fluid administration (about 6 L within the initial 72 h) than EGDT 2001(about 14 L within the initial 72 h) resulted in less mortality [24–26]. Douglas et al. divided the fluid therapy of EGDT into two phases, the early (first 6 h) and the later phases (6–72 h) and suggested that further positive fluid balance in the later phases may be counterproductive [27].

It is not clear whether fluid responsiveness guided fluid therapy improve the prognosis of critically ill patients or not. However, as fluid responders are recommended to repeat fluid therapy until responsiveness disappears, it may induce overhydration and worsen the prognosis. Furthermore, fluid challenge itself is considered to be harmful in some situations. For instance, when a patient falls into hypotension five times a day, a total of 2500 ml of fluid is required for five times 500 ml of fluid challenge [28]. For this reason, a new fluid challenge, called mini-fluid challenge, which administers a small amount of infusion in a short time has been proposed. In mini-fluid challenge, a 100 ml of colloid in 1 min [29] or 50 ml of colloid in 10 s [30] has been tried. One of the problems of this method is the need for accurate CO monitoring, since the changes in CO is small due to small preload. The most serious concern of this method is whether the results from such a small preload is reliable [28].

Instead of fluid challenge, some authors report the effectiveness of the passive leg raising (PLR) [31]. This maneuver is performed by lifting the legs and transiently increasing venous return to the intrathoracic compartment while assessing changes in CO. The advantage of PLR is that preloading is reversible and does not affect afterload, but the disadvantage is that the amount of preloading cannot be evaluated.

From these results, indication of fluid therapy needs to be judged from various examinations and physical findings.

## Conclusions

### Does Fluid Responsiveness Work?

Starling’s law explains that shifting blood from unstressed volume to stressed volume restores decreased SV/CO. Since Starling’s law does not take into consideration the decrease of afterload due to fluid-induced hemodilution, it is not an appropriate model to explain fluid responsiveness.

Like watering a plant, infusion replenishes the whole body and circulates through the whole body and does not remain in a particular compartment. Liquid taken orally is absorbed in the intestine and retained in the interstitium and then spreads from the blood vessels to the cells. As this is the case, the storage reservoir of fluid is the interstitium, not the blood vessels. It is only natural that infused solution moves rapidly from the blood vessels to the interstitial space. Thus, the effect of fluid on SV/CO is short-lived and limited.

The effect of fluid challenge on SV/CO is more complicated than we thought, and interpretation is difficult. In my opinion, fluid responsiveness based on the Starling curve would not work in the past, present, and future.

## Abbreviations

ARDS: Adult respiratory distress syndrome
CO: Cardiac output
CVP: Central venous pressure
EGDT: Early goal-directed therapy
Hb: Hemoglobin
HES: Hydroxyethyl starch
MAP: Mean arterial pressure
PVV: Pulse pressure variation
SV: Stroke volume
SVR: Systemic vascular resistance
SVV: Stroke volume variation
